# Picroside II Inhibits Neuronal Apoptosis and Improves the Morphology and Structure of Brain Tissue following Cerebral Ischemic Injury in Rats

**DOI:** 10.1371/journal.pone.0124099

**Published:** 2015-04-30

**Authors:** Tingting Wang, Li Zhao, Yunliang Guo, Meizeng Zhang, Haitao Pei

**Affiliations:** Institute of Cerebrovascular Diseases, Affiliated Hospital of Qingdao University, Shandong Provincial Collaborative Innovation Center for Neurodegenerative Disorders; Taishan Scholars Construction Project Excellent Innovative Team of Shandong Province, Qingdao, 266003, P. R. China; Federico II University of Naples, ITALY

## Abstract

This paper aimed to explore the protective effects of picroside II against the neuronal apoptosis and changes in morphology and structure that follow cerebral ischemic injury in rats. A focal cerebral ischemic model was established by inserting a monofilament thread to achieve middle cerebral artery occlusion (MCAO) in 60 *Wistar* rats, and intraperitoneal injections of picroside II (20 mg/kg) were administered. The neurobehavioral functions were evaluated with the modified neurological severity score (mNSS) test. The cerebral infarct volumes were measured with tetrazolium chloride (TTC) staining. The morphology and ultrastructure of the cortical brain tissues were observed with hematoxylin-eosin staining and transmission electron microscopy, respectively. The apoptotic cells were counted with terminal deoxynucleotidyl transferase dUTP nick-end labeling and flow cytometry, and pERK1/2 expression was determined by immunohistochemical assay and Western blot. The results indicated that neurological behavioral malfunctions and cerebral infarcts were present in the MCAO rats. In the model group, the damage to the structures of the neurons and the blood brain barrier (BBB) in the cortex was more severe, and the numbers of apoptotic cells, the early apoptotic ratio (EAR) and pERK1/2 expression were significantly increased in this group compared to the control group (*P*<0.05). In the treatment group, the neurological behavioral function and the morphology and ultrastructure of the neurons and the BBB were improved including the number of Mi increased and relative area of condensed chromosome and basement (BM) thickness descreased, and the cerebral infarct volume, the number of apoptotic cells, the EAR and pERK1/2 expression were significantly decreased compared to the model group (*P*<0.05). These results suggest that picroside II reduced apoptosis and improved the morphology and ultrastructure of the neurons and the BBB and that these effects resulted in the recovery of the neurobehavioral function of rats with cerebral ischemia.

## Introduction

Ischemic cerebrovascular disease is a common neurological disease. Cerebral ischemia can trigger neuronal apoptosis in the cerebral ischemic penumbra; however, this process is potentially reversible[1]. Early studies demonstrated that brain tissue that is deprived of oxygen following cerebral ischemia and neuronal injury exhibits necrosis or apoptosis and that the extracellular signal-regulated kinase 1/2 (ERK)1/2 signaling pathway is simultaneously activated to facilitate cell proliferation, differentiation and repair, which alleviates the damage to nerve cells[2–3]. However, an increasing number of experiments have shown that cerebral ischemia activates the ERK1/2 pathway to accelerate the inflammatory response, which leads to neuronal apoptosis[4]. Deng *et al*. [5] reported that the ERK1/2 pathway inhibitor U0126 and the calcium channel blocker ketamine can suppress ERK1/2 phosphorylation, block miRNA-21 and matrix metalloproteinase-9 (MMP-9) expression and inhibit the inflammatory response to relieve the damage to the blood-brain barrier (BBB) and brain edema. Our research group has proven that picroside II plays multiple roles in protecting brain tissue from cerebral ischemic injury, including mediating anti-oxidative and anti-inflammatory effects, relieving brain edema and suppressing neuronal apoptosis [6–11]; however, cell morphology and structure are still the most reliable indices for evaluating the damage to and repair of the nervous system. Thus, in the present work, we examined the influence of picroside II on neuronal apoptosis, morphology and structure following cerebral ischemic injury to further verify the neuroprotective role of picroside II in cerebral ischemia.

## Materials and Methods

### 1.1 Ethics Statement

This experiment was approved by the Ethics Committee of Qingdao University Medical College (QUMC 2011–09). The local legislation regarding the ethics of animal experimentation and the guidelines for the care and use of laboratory animals were followed in all animal procedures. All surgeries were performed under chloral hydrate anesthesia, and all efforts were made to minimize suffering.

### 1.2 Animal models

A total of 60 healthy adult male specific pathogen-free (SPF)-grade *Wistar* rats weighting 220–250 g were purchased from the Experimental Animal Center of the Qingdao Drug Inspection Institute (SCXK [LU] 20120010). All animals were housed at an ambient temperature of (25±2°C with natural illumination and free access to food and water for 7 days to adapt to the environment. Fifteen of these rats were randomly chosen as the control group, and the remaining 45 rats were used to establish middle cerebral artery occlusion (MCAO) models via the insertion of a monofilament suture into the middle cerebral artery [12–13]. All rats were fasted prior to the operation, anesthetized with intraperitoneal injections of 10% chloral hydrate (3 ml/kg), fixed in the supine position and subjected to an aseptic operation. A monofilament suture was intraluminally introduced from the left external carotid artery into the internal carotid artery to block the MCA for 2 h. After 2 h, the modified neurological severity score (mNSS) test [14] was used to evaluate the neurological functions of the rats, and the rats with mNSS scores of 7–12 were regarded as successful models; those that did not meet this standard or died were removed from the experiment. Ultimately, 30 successful rat models were randomly divided into model and treatment groups consisting of 15 rats each. The rats in the control group were underwent the same surgical procedures with the exception of the insertion of the monofilament to cause occlusion.

### 1.3 Intervention

Picroside II was supplied by Tianjin Kuiqing Med. Tech. Co. Ltd. (CAS No: 39012-20-9, purity> 98%, molecular weight: 512). The picroside II was diluted to a 1% solution with normal saline. Two hours after the establishment of MCAO, the animals in the treatment group were administered an intraperitoneal injection of picroside II (20 mg/kg) [15]; the animals in the control and model groups were injected with the same volume of normal saline.

### 1.4 Evaluation Index

#### 1.4.1 Neurobehavioral function test

All animals were subjected to the mNSS test to assess their neurobehavioral function before treatment and at 24 h after treatment. Higher scores on this test indicate greater neurological behavioral impairments. Multi-group comparisons were made with repeated measures analyses of variance (ANOVA) and multiple comparison among different groups and different time pairwise was made with multivariate ANOVA.

#### 1.4.2 Tetrazolium chloride (TTC) staining

At 24 h after intervention treatment, 5 rats were randomly chosen from each group and were anesthetized with 10% chloral hydrate (300 mg/kg), perfused with 200 ml of normal saline from heart into the aorta, decapitated at cervical vertebrae and then underwent craniotomy to remove the brain completely. The brain tissue was cut into five 2-mm thick sections in the coronal plane, which were stained with a 2% solution of TTC phosphate buffer for 10 min at 37°C away from light. The normal brain tissue appeared uniformly red, while the cerebral infarct zones appeared white. Images of the stained sections were taken and analyzed using Adobe Photoshop CS to calculate the cerebral infarct volumes (CIVs). The CIVs are expressed as percentages of the infarct area / the area of the ipsilateral hemisphere at the coronal section of the optic chiasma.

#### 1.4.3 HE staining

At 24 h after treatment, 5 rats were randomly selected from each group and anesthetized with 10% chloral hydrate and then perfused with 200 ml of 4% formaldehyde via the heart. The brains were removed and fixed in 4% formaldehyde for 2 h, soaked in distilled water for 4 h, dehydrated using a graded ethanol series, hyalinized via dimethylbenzene, embedded in paraffin, and sectioned at a thickness of 5 μm. The sections were adhered to glass slides treated with poly-L-Lysine, which were stored at 4°C. After routine de-waxing and washing, the histological sections were stained with hematoxylin for 5 min, the color was separated with 1% hydrochloric acid alcohol for 20 s, the section were back to blue with 1% ammonia for 30 s, dyed with eosin for 5 min, dehydrated in increasing concentrations of alcohol, hyalinized with dimethylbenzene, and sealed with neutral gum. Under a microscope at 400X magnification (Leica DMI400, Germany), 5 non-overlapping views of the cortex in each section were randomly chosen, and the cells in these sections were counted and presented as mean ± SD. The denatured cell index (DCI = denatured cell number/total cell number) was used to indicate the degree of damage.

#### 1.4.4 Terminal deoxynucleotidyl transferase dUTP nick end labeling (TUNEL) assay

Paraffin sections treated as above were deparaffinized with dimethylbenzene, hydrated with in a gradient of ethanol and washed with distilled water according to the manual for the TUNEL apoptosis detection kit (KGA7025-50 assays, KeyGEN Biotech. Co. Ltd., Nanjing, China). DNase I (5000 U) was added to some of the paraffin sections, which were regarded as the positive samples, and those without TdT were regarded as the negative samples. Under a microscope at 400X magnification (Leica DMI400, Germany), we found that the nuclei of the apoptotic cells in the positive samples appeared brown, and no such nuclei were present in the negative samples. Five non-overlapping fields in the cortex of each section were randomly observed, and the positive cells in these fields were counted. The results are presented as mean ±SD. The apoptotic cell index (ACI = apoptotic cells / total cells) was calculated and used as an indicator of the degree of apoptosis.

#### 1.4.5 Immunohistochemical staining

Rabbit anti-rat pERK1/2 monoclonal antibody (1:100, # 4370) was purchased from Cell Signaling Tech. Co. Ltd., USA. Rabbit anti-rat ERK1/2 polyclonal antibody (1:100, BS-2637R) was provided by Bioss Biotech. Co. Ltd., Beijing, China. 3,3’-diaminobenzidine (DAB) and Power Vision two-step histo-staining reagent (PV-6001) were obtained from ZSGB-Bio. Co. Ltd., Beijing, China. Paraffin sections treated as above were de-waxed and washed according to routine procedures. The immunohistochemical procedures were performed in strict accordance with the manufacturer’s instructions. Under a microscope, the positive cells appeared with brown granules in the cytoplasm. Those sections to which 0.01 mmol/L PBS was added instead of primary antibody exhibited no positive responses. The positive cells were enumerated and averaged in five random views of cortex from four serial slices under a microscope at 400X magnification (Leica DMI400, Germany) The positive cell index (PCI = positive cells / total cells) was calculated and used to indicate the pERK1/2 and ERK expression levels.

#### 1.4.6 Flow cytometry (FCM)

Five rats in each group were anesthetized and decapitated at the given time points above. The brains were completely removed and placed on ice, and 200 mg of brain tissue was then cut from the ischemic cortex and placed in a 1.5 ml EP tube. After added 0.5 ml of precooled PBS, the brain tissue were cut into pieces, placed in a glass tube, and incubated with 1.5 ml 2.5% EDTA free trypsin at 37°C for 15 min. Next, the sample was gently blown through a straw to filter into a 1.5 ml EP tube with a 200 mesh strainer (on ice) and was centrifuged at 12,000 r/min for 5 min at 4°C. The supernatant was then discarded, and the cell concentration was adjusted to 1×10^6^/100 μl with 1×Annexin binding buffer. The sample was then dyed with an Annexin V-FITC apoptosis detection kit (KeyGEN Biotech. Co. Ltd., Nanjing, China). A FACScan Calibur (Becton Dickinson Medical Devices Co. Ltd., USA) system was used to perform FCM within 1 h according to the following procedure: 100 μl of FCM suspension was taken into an EP tube, 5 μl of Annexin V (AV) and 1 μl of 1 iodide (PI) were added, the tube was left at room temperature for 15 min, 500 μl of PBS was added, and the contents were filtered into a glass tube with a 200 mesh strainer (on ice). The FCM instrument was then used to detect the percentage of apoptotic cells with an excitation wavelength of 488 nm and emitting wavelengths of 535 nm and 575 nm. FlowJo 7.6 was used to analyze the early apoptotic ratio (EAR), the results of which are expressed as mean ±SD.

#### 1.4.7 Western blot analysis

at 24 h after treatment, 100 mg of ischemic brain tissue from each rats as above was collected and placed into 1.5 ml Eppendorf tubes. Then, 1 ml of cell lysis buffer was added at a proportion of 10 mg:100 μl (1 ml + 10 μmol/L PMSF, No. P0013B, Biyuntian Biotech. Co. Ltd., China). The cells were ground at 4°C in an ice bath, completely lysed with gentle shaking at 4°C for 30–60 min, centrifuged at 12,000 r/min for 15 min at 4°C (Eppendorf 5801, Germany), and the supernatant was collected into a fresh Eppendorf tube. Protein concentrations was determined using an enhanced BCA protein assay kit (No. P0010, Biyuntian Biotech Ltd., China). Protein samples were mixed with 5X SDS-PAGE sample loading buffer, followed by denaturation at 95–97°C for 5 min, and stored at -80°C until further analysis. A sample containing 20μg total protein was separated by 10% PAGE gels, and transfered to polyvinylidene difluoride (PVDF) membranes.The PVPF membrane was blocked with 5% v/v nonfat milk in Tris-buffered Saline/Tween-20 (TBST) buffer (20 mM Tris–HCl, pH 7.6, 136 mM NaCl and 0.1% v/v Tween-20) and then probed for overnight at 4°C with primary antibodies: pERK1/2, 1:2000. The membranes was incubated with goat anti-rabbit HRP-conjugated secondary antibody (1:10000, AB136817, Abcam, USA) for 1 h with gentle agitation at room temperature. Protein signals were visualized using Immobilon^TM^ Western chemiluminescent HRP substrate (Millipore, USA). Images were acquired by a BioSpectrum 810 imaging system (UVP, USA) and analysed using the Quantity One software.The membranes were immersed in Restore Western blot stripping buffer (21062, Thermo, USA) for 10 min to remove the first antibody, subsequently washed in TBST, blocked for 60 min, and incubated with rabbit anti-rat ERK1/2 monoclonal antibody (1:1000, # 9102, Cell Signaling Technology, USA) at 4°C overnight with gentle shaking. The blot was visualised and analysed as described above. The relative value of the protein (RVP) = the grey value of pERK1/2/the grey value of ERK1/2. The experiment was repeated 5 times, and the results are presented as the means ± standard deviation.

#### 1.4.8 Transmission electron microscopy (TEM)

A small amount of fresh brain tissue was taken from the ischemic cortex, cut into small pieces of 1 mm×1 mm×1 mm, fixed in 2.5% glutaraldehyde for 24 h and rinsed with PBS for 10 min 3 times. The sample was then post-fixed in osmic acid for 1 h, rinsed with PBS for 5 min 3 times, stored at 4°C overnight, dehydrated using graded acetone, and embedded with Epon 812 epoxy resin. An ultra-microtome (Leica EM UC6, Germany) was used to cut the tissue into 50-nm ultrathin slices. The slices were then placed on a copper grid prepared with polyvinyl formal and stored at 4°C. 3% uranyl acetate-alcohol saturated solution and 6% lead citrate dye liquor were prepared for the next steps. The copper grid of ultrathin slices was covered with a petri dish onto which a drop of 3% uranyl acetate-alcohol saturated solution was placed (pH 3.5). The ultrathin slices were exposed to the dye liquor, dyed for 30 min, rinsed with double-distilled water for 10 min 3 times, and sucked dry of their moisture contents. Using the same method, a drop of 6% lead citrate dye liquor (pH 12) was placed in another petri dish, and the slices were exposed to this liquor for 5 min of dying, followed by rinsing in carbon dioxide-free double-distilled water 3 times for 10 min each. The slices were then drained of water and dried at room temperature. Finally, the ultrastructures of the neurons and the BBB were observed under TEM (JEM-1200EX, Japan). Five different neurons in the cortex of each section were randomly observed, and the relative area expressed as percentages of the condensed chromosome (CC) area / the total nuclear area and the number of mitochondria (Mi) were counted. Five non-overlapping views of BBB were randomly observed, and the basement membrane (BM) thickness and the relative thickness which expressed as the BM thickness / the endothelium thickness were counted. The results are presented as mean ±SD.

### 1.5 Statistical analysis

The SPSS 17.0 software was used for the statistical analyses. Results were expressed as mean±SD($\bar{x}$±s). Multi-group comparisons were made with one-way analyses of variance (one-way ANOVAs), and two-group comparisons were made with Bonferroni tests. The values were considered to be significant when *P* was less than 0.05.

## Results

### 2.1 Neurobehavioral functional evaluation (Table 1)

The rats in the control group exhibited normal neurobehavioral function, while those with MCAO exhibited neurobehavioral malfunction. The ANOVA for repeated measures showed that the treatment effect, time effect and the interaction between treatment effects and time effects were significant (*F* = 211.31~785.42, *P*<0.05). The results of multiple comparison tests indicated that the mNSS scores of the model group increased with the time prolonging (*P*<0.05), but the difference between before and after treatment was no signigicant (*P*>0.05). Before treatment, there was no significance between model and treatment groups (*P*>0.05). After treatment, the mNSS scores of treatment group was obviously lower compared to model group (*P*<0.05).

**Table 1:** Modified neurological severity score points(Mnss).

Group	n	Before MCAO	Before Treatment		After Treatment
Control group	15	0	0	0	
Model group	15	0	10.00±1.65*	10.53±1.64*	
Treatment group	15	0	9.93±1.33*	7.46±1.41* ^#^△	

At the same time,

*P<0.05, compared with control group

#*P*<0.05, compared with model group

△*P*<0.05, self-compared between before and after treatment

### 2.2 Cerebral infarct volume (CIV) (Fig 1)

**Fig 1:**
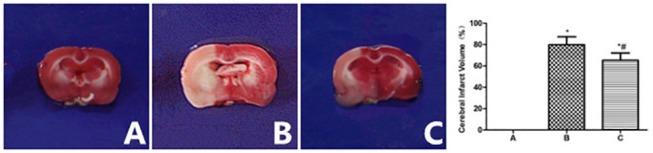
Cerebral Infarct Volume in each group, TTC staining. A. Control group; B. Model group; C. Treatment group; *P<0.05, compared with control group; #*P*<0.05, compared with model group

TTC staining revealed that the brain tissue of the control group exhibited a uniformly red color that indicates that no infarcted areas were present, while large CIVs (79.82±7.56) were observed in the model group. The treatment group (65.32±6.90) exhibited smaller CIVs than the model group (*t* = 3.88, *P*<0.05, Fig 1). We shown a complete brain before TTC staining (S1 Fig).

### 2.3 Histological changes (Fig 2)

**Fig 2:**
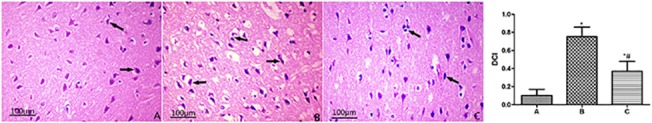
The morphology and structure of nerve cells in cortex in different group, HE×400. A. Control group; B. Model group; C. Treatment group; →. Denatured cells *P<0.05, compared with control group; #*P*<0.05, compared with model group

HE staining revealed that the neurocyte bodies in the cortex of the control group were larger, and the nuclei exhibited basophilic blue staining, while the cytoplasm appeared as acidophilic red. The nerve cells arranged well, with complete structure and even color. In the model group, the nerve cells arranged irregularly, the nuclei were condensed and deeply dyed, the majority of the nerve cells had changed to vacuoles after degeneration and necrosis, and the DCI (0.75±0.11) of this group was obviously higher than that of the control group (0.10±0.07) (*t* = 11.71, *P*<0.05). The damage to the nerve cells in the treatment group was less severe, and the DCI in this group was 0.37±0.11, which was much lower than that of the model group (*t* = 6.91, *P*<0.05, Fig 2).

### 2.4 Ultrastructures (Figs 3–6 and S1 Table)

**Fig 3:**
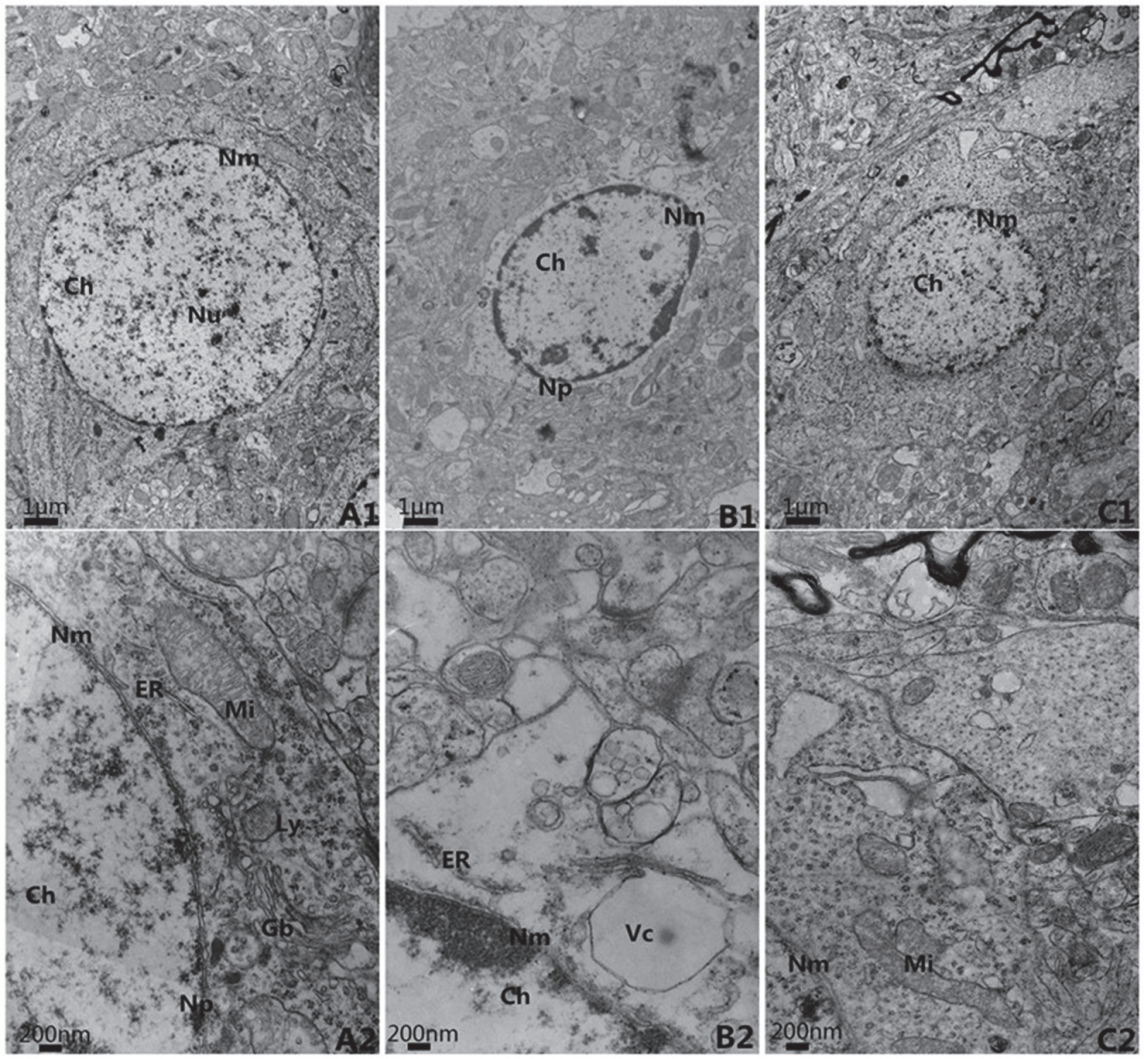
The ultra-structure of neuron in cortex, EM×5k, 20k. A. Control group; B. Model group; C. Treatment group Nu: Nucleolus; Np: Nuclear pore; Nm: Nuclear membrane; Ch: Chromatin; Mi: Mitochondria; ER: Endoplasmic reticulum; Gb: Golgi bodies; Ly: Lysosome; Vc: vacuole

In the control group, the neurons in the cortex were clearly observable and had large round nuclei, even chromatin and clear cytoplasm (Fig 3A1). At high power, the double nuclear membranes were clear and complete, the outer membrane enclosed the periphery of the mitochondria, the inner layer was enfolded to form cristae, different-sized vesicles existed in the Golgi bodies, and the cell organelles, such as the lysosome, endoplasmic reticulum, etc., were distributed throughout the cytoplasm (Fig 3A2). The vascular endothelial cells had smooth and flat surfaces, and the endothelia, basement membranes and foot processes were in close contact (Fig 5A1 and 5A2). In the model group, the neurons in the cortex were irregular and exhibited chromatin condensation, cytoplasm dissolution and vacuole formation (Fig 3B1). Compared with control group, the relative area (9.18±0.72) was significantly higher(*t* = 21.59,*P*<0.05), and the number of Mi (1.2±0.84) was obviously lower (*t* = 13.51,*P*<0.05). At high power, the nuclear membranes and cell organelles were dissolved or absent (Fig 3B2). The glial cells around the vacuoles had dissolved and disappeared, tube invagination was present, and the inner surfaces of the blood vessels were rough (Fig 5B1). At high power, the endothelial cells were swollen, and the thickened basement membrane was not well organized (Fig 5B2). The BM thickness and relative thickness significantly increased than those in control group (*t* = 15.40,5.72,*P*<0.05). In the treatment group, the damage to the neurons was alleviated (Fig 3C1 and 3C2) compared to the model group. The relative area (1.80±0.55) was much lower than model group (*t* = 19.82, *P*<0.05) and the number of Mi (5.8±0.84) was much higher (*t* = 9.14, *P*<0.05). The vascular endothelial cells and the basement membrane exhibited smooth and intact surfaces with clear layers (Fig 5C1 and 5C2). BM was much narrower than model group (*t* = 12.56, *P*<0.05), but there was no significance on relative thickness (*t* = 1.82, *P*>0.05).

**Fig 4:**
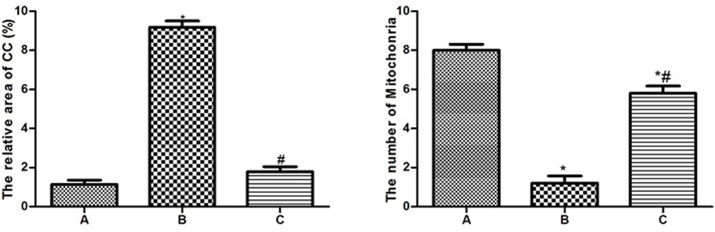
The relative area of condensed chromosome and number of Mitochongria in neurons. A. Control group; B. Model group; C. Treatment group *P<0.05, compared with control group; #*P*<0.05, compared with model group

**Fig 5:**
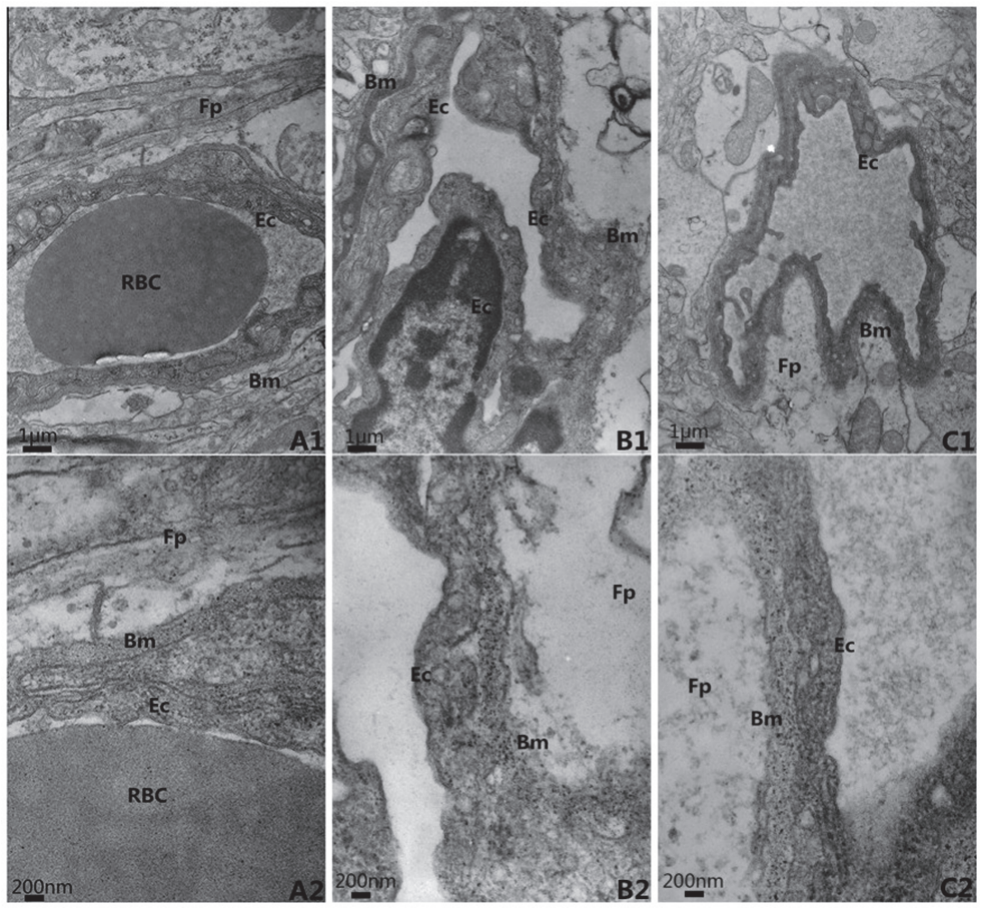
The ultra-structure of blood brain barrier, EM×20k, 60k. A. Control Group; B. Model group; C. Treatment group Bm: basement membrane; Ec: endothelial cell; Fp: foot process; RBC: red blood cell A. Control Group; B. Model group; C. Treatment group Bm: basement membrane; Ec: endothelial cell; Fp: foot process; RBC: red blood cell

**Fig 6:**
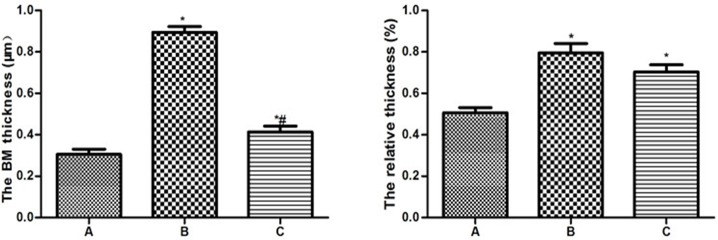
The basement thickness and the relative thickness in BBB. A. Control group; B. Model group; C. Treatment group *P<0.05, compared with control group; #*P*<0.05, compared with model group

### 2.5 Neuronal apoptosis (Fig 7)

TUNEL assays revealed few apoptotic cells in the control group (ACI = 0.11±0.07). The ACI of the model group (0.59±0.10) was significantly elevated compared to the control group (*t* = 9.16, *P*<0.05); in the model group, the nerve cells arranged irregularly, the cell bodies were shrunken, and the gaps around the cells had increased in size. The ACI (0.33±0.07) was statistically lower in the treatment group than in the model group (*t* = 5.03, *P*<0.05).

**Fig 7:**
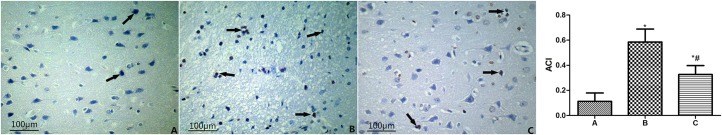
Apoptosis in cortex of rats, TUNEL×400. A. Control group; B. Model group; C. Treatment group; →. Apoptotic cells * *P*<0.05, compared with control group; #*P*<0.05, compared with model group

### 2.6 Early apoptotic ratio (EAR) (Fig 8)

**Fig 8:**
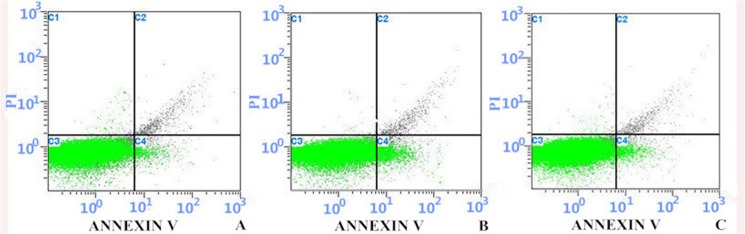
Early Apoptosis Percentage determined by Flow cytometry, Annexin V-FITC staining. A. Control group; B. Model group; C. Treatment group FlowJo7.6 software was applied to analyze the early apoptosis percentage (C4 quadrant)

Flow cytometry showed that EAR in the control group kept a lower level (1.47±0.33)while it reached a peak of 5.44±0.40 in the model group, markedly higher than that in control group (t = 14.18, P<0.05). In treatment group, EAR dropped to 3.43±0.56, and it exists statistical difference as compared with the model group (t = 7.16, P<0.05, shown in Fig 8).

### 2.7 Immunohistochemistry (Fig 9)

**Fig 9:**
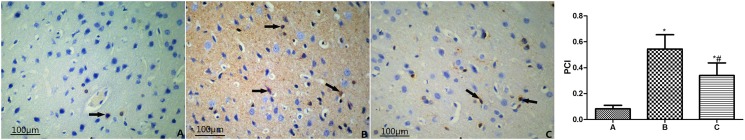
The expression of pERK1/2 in cortex of rats,DAB×400. A. Control Group; B. Model group; C. Treatment group; →. Positive cells * *P*<0.05, compared with control group; #*P*<0.05, compared with model group

In the control group, the cells were lightly dyed, and pERK1/2 expression was significantly weaker (PCI = 0.08±0.03) compared to the model group (PCI = 0.54±0.11) (*t* = 8.47, *P*<0.05). The PCI in the treatment group (0.34±0.10) was obviously lower than that in the model group (*t* = 3.74, *P*<0.05, Fig 9).

### 2.8 Western Blot (Fig 10)

**Fig 10:**
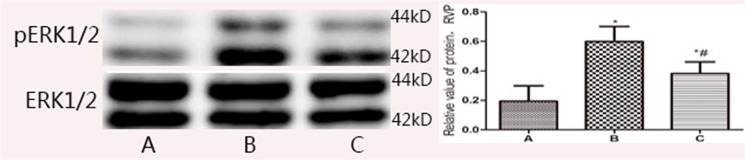
The expression of pERK1/2 determined by Western blot. A. Control Group; B. Model group; C. Treatment group; * *P*<0.05, compared with control group; #*P*<0.05, compared with model group

Western blot shown that the pERK1/2 expression in model group (RVP = 0.60±0.10) was significantly higher than in control and treatment groups (*t* = 6.641, 3.553, *P*<0.05). pERK1/2 expression in the control group, which RVP was 0.38±0.08, was much lower compared to model group, but was still higher than that in control group (RVP = 0.19±0.11, *t* = 3.087, *P*<0.05, shown in Fig 10).

## Discussion

Within minutes of the onset of acute ischemic stroke, the regional cerebral blood flow (rCBF) decreases dramatically, and the nerve cells are damaged; this damage is characterized by necrosis or apoptosis. During this process, the ERK1/2 signaling pathway is activated by growth factors, oxidative stress, increases in intracellular Ca^2+^ levels, and other factors to regulate various physiological processes, including cell proliferation, differentiation and apoptosis[2]. ERK1/2 activation can induce cell apoptosis via both the intrinsic and extrinsic pathways[1, 16]. Certain stressors can active the intrinsic pathway to release mitochondrial cytochrome c and to activate cysteine-containing aspartate-9 (caspase-9)[17]. The extrinsic pathway is primarily activated by the activation of receptors for compounds including tumor necrosis factor alpha (TNFα), Fas (Apo-1, CD95), caspase-8, etc. [18]. Namura *et al*.*’s* [19] research showed that U0126 has a neuroprotective effect that reduces cerebral infarct volumes following forebrain ischemia and focal cerebral ischemia; UO126 also protects mouse primary cultured cortical neurons from oxygen deprivation for 9 h and protects these cells from nitric oxide toxicity. Additionally, it has been suggested that ERK1/2 activation induces neuron swelling or necrosis independent of caspase-3 activation[20].

Picroside II is the principal active component of *Picrorhiza*, which is a traditional Chinese medicine. Our previous research demonstrated that, following brain ischemic injury, picroside II can remove free radicals, has antioxidant properties, decreases MMP-9 expression and inhibits the toll-like receptor 4 and nuclear factor kappa B (TLR4-NFκB) signaling pathways to relieve cerebral edema. Moreover, picroside II down-regulates caspase-3 and poly ADP-ribose polymerase (PARP) expression to inhibit the cell apoptosis that is induced by cerebral ischemic reperfusion injury[6–11]. The best therapeutic dose and time window were intraperitoneal injections of picroside II at a dose of 10–20 mg/kg body weight following cerebral ischemia by 1.5–2.0 h [21–22]. Our experimental results suggest that ERK1/2 was activated following cerebral ischemia, which would induce cell apoptosis and damage to the neurons and BBB and would thus increase the cerebral infarct volume and cause neurobehavioral dysfunction. After treatment with picroside II, pERK1/2 expression was decreased, and both cell apoptosis and the damage to the ultrastructures of the neurons and BBB were reduced including the number of Mi increased and relative area of condensed chromosome and basement (BM) thickness descreased; thus, the cerebral infarct volume was diminished, and the mNSS scores were reduced. These findings further suggest that cerebral ischemia can induce cell apoptosis; however, picroside II might reduce cell apoptosis and improve the morphologies and ultrastructures of neurons andthe BBB to help to recover the neurobehavioral function of rats with cerebral ischemia.

## Conclusion

Our findings suggest that picroside II might reduce apoptosis and improve the morphologies and ultrastructures of neurons and the BBB to help to recover the neurobehavioral function of rats with cerebral ischemia.

## Supporting Information

S1 Fig The brain of rats following MCAO. The right hemisphere shown uniform pink, while MCAO caused the ischemic injury of the left hemisphere which shown pale with the hemisphere swelling.
(TIF)

S1 Table Different Evaluation Index of Ultrastructures. *P<0.05, compared with control group; #*P*<0.05, el group
(XLS)

S2 Table Different Evaluation Index. *P<0.05, compared with control group; #*P*<0.05, compared with model group
(XLS)
