# Supplementary figures and images for: Picroside II Inhibits Neuronal Apoptosis and Improves the Morphology and Structure of Brain Tissue following Cerebral Ischemic Injury in Rats
Source: PLoS One. 2015 Apr 30;10(4):e0124099. doi: 10.1371/journal.pone.0124099 (PMC4415915; doi:10.1371/journal.pone.0124099)

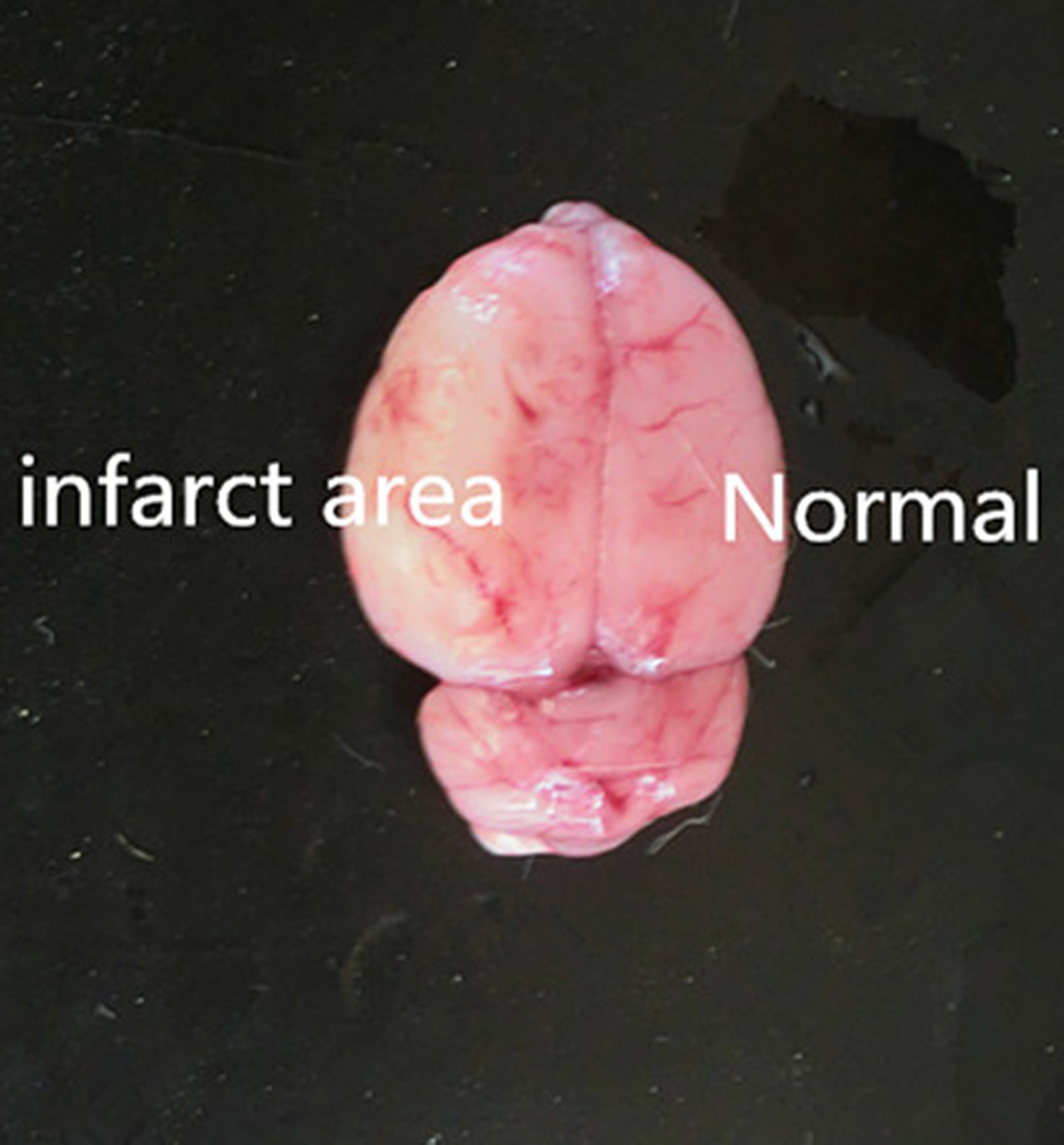

Supplement: S1 Fig — The right hemisphere shown uniform pink, while MCAO caused the ischemic injury of the left hemisphere which shown pale with the hemisphere swelling. (TIF) [file pone.0124099.s001.tif]
